# The effectiveness of a novel surgical approach for the treatment of congenital muscular torticollis

**DOI:** 10.1097/MD.0000000000019572

**Published:** 2020-04-03

**Authors:** Jianlin Shan, Heng Jiang, Yang Zhang, Zhicheng Zhang, Dajiang Ren, Bing Zhu

**Affiliations:** a Department of Orthorpaedics, Beijing Friendship Hospital, Capital Medical University; b Department of Orthorpaedics, PLA Army Genertal Hospital, Beijing; c Department of anatomy, Chengdu Medical College, Chengdu, China.

**Keywords:** anatomy, cervical fascia, congenital muscular torticollis, sternocleidomastoid muscle

## Abstract

The outcomes of surgical treatment for congenital muscular torticollis (CMT) are poor in many cases, primarily from surgeon's fear of damage to important blood vessels in the carotid sheath. The current research aimed to establish a novel, safer and more effective surgical approach for the treatment of CMT based on the anatomic relationship between the fascial sheath of the sternocleidomastoid muscle and the carotid sheath.

A total of 12 formalin-fixed cadaveric specimens (including 9 males and 3 females with average age of 48.1 years at the time of death) were used to observe the anatomical integrity and the compactness of the sternocleidomastoid fascial sheath and its relationship with the carotid sheath. From January 2013 to December 2015, 22 patients with CMT were treated surgically, including 12 males and 10 females with an average age of 13 years (range: 6–28 years). All patients underwent surgical treatment using the novel approach designed by the author, that is, separation of the fascial sheath of the sternocleidomastoid and the carotid sheath was performed.

The fascial sheath of the sternocleidomastoid muscle is easily isolated from the carotid sheath in all specimens. All procedures were successfully completed. The space between the fascial sheath of the sternocleidomastoid muscle and the carotid sheath can be accessed in all patients. Carotid sheath contracture was identified in 1 patient and divided successfully. The average follow-up period was 19 months (range: 5 to 43 months). No postoperative lateral band was noted in the neck in any of the patients. Satisfaction regarding cosmetic and functional outcomes was considered excellent in 13 patients, good in 7 patients, and fair in 2 patients.

The fascial sheath of the sternocleidomastoid muscle is easily isolated from the carotid sheath. This anatomical feature is the basis of division of the sternocleidomastoid muscle and can improve the safety and effectiveness of surgical treatment for CMT in patients older than 6 years old.

## 1 Introduction

Congenital muscular torticollis (CMT) is a common ailment in children and juveniles, with an incidence of 0.4% to 3.92%.^[1–4]^ Most children with CMT can improve with nonoperative treatment before 1 year of age, and surgical treatment should be considered if the efficacy is poor. The optimal timing for surgery is believed to be at an age between 1 and 4 years.^[5–11]^ Unfortunately, many patients receive surgical treatment in adulthood for various reasons. While most patients are satisfied with the surgical outcomes, a considerable number of patients are not. The factors negatively affecting treatment efficacy mainly include the following:

(1) The timing of surgical treatment is too late because head, face, and cervical deformities have already formed.^[4,5,12–14]^
(2) Tissue contractures were not completely released during correction.^[1,4,6]^

The former cannot be changed by the surgeon, but the latter can likely be improved by the surgeon's efforts. A postoperative lateral band is a manifestation of incomplete release of a tissue contracture, with an incidence rate as high as 38% to 75% according to the literature, and affects the efficacy of surgical correction.^[4,5,13,15,16]^ Although postoperative lateral bands are associated with a surgeon's skill, this is not the focus of this study. This study aimed to investigate inherent factors causing lateral bands in the current surgical management of CMT and to focus on techniques to overcome them.

The rationale for any surgical approach depends first and foremost on whether it is suitable for the indicated pathological anatomy. Since the sternocleidomastoid muscle is enveloped by the investing fascia, the pathological changes associated with CMT include not only contracture of the sternocleidomastoid muscle but also contracture of its fascial sheath. Depending on the severity and duration of the disease, contracture of the posterior carotid sheath and the scalene muscle may be involved. Therefore, CMT correction may require management of the above structures in addition to division of the sternocleidomastoid muscle.^[5,6,17–19]^

Various surgical approaches are available for CMT treatment, with unipolar sternocleidomastoid release as the most common procedure. In conventional unipolar sternocleidomastoid release, the fascial sheath of the sternocleidomastoid is opened first to isolate and divide the contracture of the sternocleidomastoid muscle. Then, the procedure is continued for any remaining lateral bands,^[5,17,19,20]^ which are often identified as the sternocleidomastoid fascia posterior sheath and/or the carotid sheath.^[6]^ Since the fascial sheath of the sternocleidomastoid is immediately adjacent to the carotid sheath, the contents within the carotid sheath, especially the internal jugular vein, can be damaged during division of residual tension bands anterior to the carotid sheath. Accordingly, although surgical correction of CMT is a minor operation, possible severe complications during surgery should be considered. In the conventional surgical procedure, the surgeon may not divide all bands causing CMT because of safety concerns. If surgical safety cannot be guaranteed, surgeons often prefer performing incomplete correction. As a result, postoperative lateral bands may be present. Therefore, to avoid damaging the contents within the carotid sheath, surgeons may not completely divide the posterior sheath of the sternocleidomastoid muscle fascia, and contracture of the posterior structure is possible, which may account for incomplete correction.

Considering the importance of completely dividing the posterior sheath of the sternocleidomastoid muscle for effective CMT correction, the author observed the anatomical relationship between the fascial sheath of the sternocleidomastoid and the carotid sheath and designed a novel approach for the treatment of CMT. Anatomical observation in cadavers and clinical practical application revealed the feasibility and safety of the novel approach, which showed a significant advantage in reducing or eliminating postoperative bands compared with conventional surgical approaches.

## 2 Materials and methods

### 2.1 Anatomical studies

**Figure 1:**
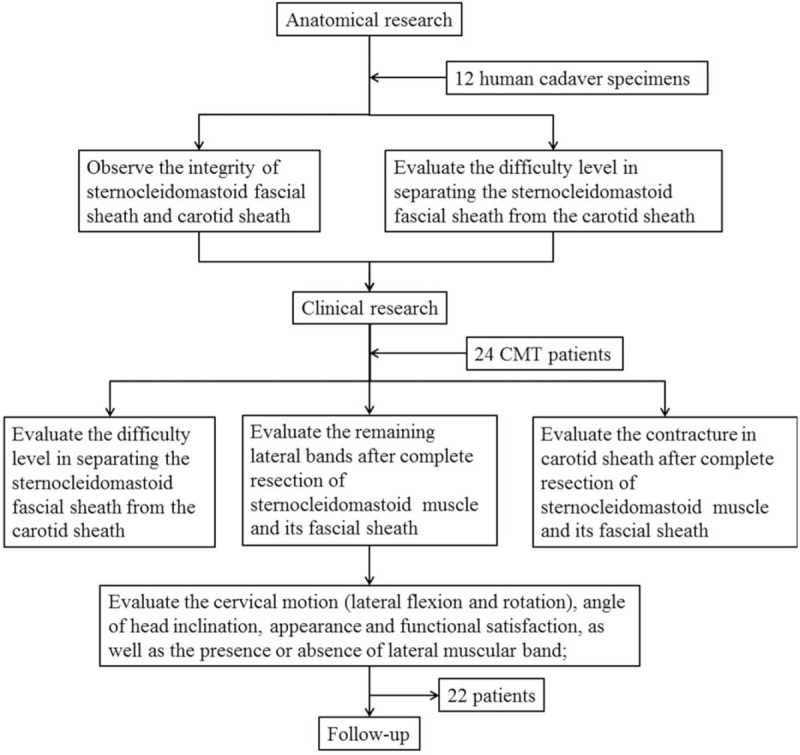
The flow chart of the current study.

The flow chart of current study was shown in Fig. 1.

A total of 12 human cadaver specimens (including 9 males and 3 females with a mean age of 48.1 years (range: 15 to 61 years) at the time of death) fixed in formalin were selected. Specimens with a history of trauma or surgery in the neck and lesions on gross examination were excluded.

(1) Relationship between the investing fascia and the platysma muscle: an incision was made to cut the skin and platysma muscle along the clavicle. Careful separation was performed inferiorly to superiorly between the platysma muscle and the investing fascia to free the platysma and the skin until the level of the hyoid bone. The integrity of the investing fascia was maintained during separation. According to the relationship between the investing fascia and the platysma muscle, the magnitude of difficulty in stripping the platysma muscle from the superior surface of the investing fascia was evaluated.
(2) Relationship between the fascial sheath of the sternocleidomastoid and the carotid sheath: dissection was performed in the left and right sides of each cadaver specimen. The investing fascia was incised 2 cm longitudinally, 0.5 cm medial to the medial edge of the sternocleidomastoid muscle (without damaging the fascial sheath of the sternocleidomastoid); the incision was centered 1.5 cm above the clavicle. First, primary separation was performed along the deep surface of the sternocleidomastoid muscle sheath with a nerve stripper, followed by further separation using an index finger on the deep surface of the sternocleidomastoid muscle sheath to the lateral side of the sternocleidomastoid. Then, the incision was extended superiorly and inferiorly to 3 cm in length. During separation, the degree of difficulty of separation and the integrity and compactness of the investing fascia and the carotid sheath were evaluated. The sternal head and the clavicular head of the sternocleidomastoid muscle were divided approximately 1.5 cm above the clavicle. The divided ends were retracted superiorly and inferiorly to further observe the integrity and compactness of the investing fascia and the carotid sheath.

### 2.2 Patients and methods

The present study was conducted in accordance with the Declaration of Helsinki and was approved by the Ethics Committee of PLA Army Genertal Hospital. Written informed consent was obtained from all participants.

From January 2013 to December 2015, 24 patients with CMT were admitted and treated in our hospital. The follow-up was completed in 22 patients, including 12 males and 10 females with an average age of 13 (6–28) years. The ages of 13 patients were between 6 and 12 years and the ages of 9 patients were over 12 years. Right CMT was diagnosed in 11 patients, left CMT was diagnosed in 10 patients, and bilateral CMT was diagnosed in one patient. All patients underwent the procedure described below, which was completed by the author.

### 2.3 Operative technique and postoperative care

**Figure 2:**
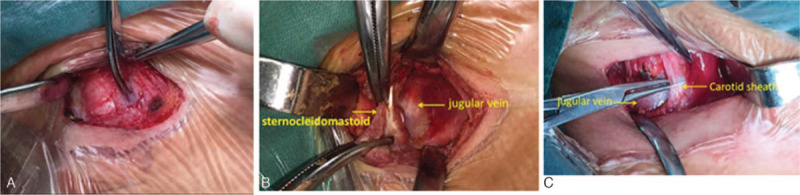
The novel surgical approach for the treatment of CMT. Fig. 2-A shows that the investing fascia was incised at the medial edge of the sternocleidomastoid muscle, and the intact anterior part of the fascial sheath of the sternocleidomastoid can be visualized. As shown in Fig. 2-B, the sternocleidomastoid muscle was incised after separation between the sternocleidomastoid fascial sheath and the carotid sheath. Fig. 2-C shows that the incised sternocleidomastoid muscle and its fascial sheath are naturally contracted distally and proximally; posterior contracture of the carotid sheath is also shown.

The patient was placed in the supine position with the head and neck tilted to the contralateral side and slightly extended to the posterior side. An approximate 4-cm incision was made 1.5 to 2 cm above and parallel to the clavicle. The skin and subcutaneous tissue were incised. The platysma muscle was incised along the incision. The deep surface of the platysma muscle was separated and retracted superiorly and inferiorly to visualize the distal end and the tendon of the sternocleidomastoid muscle. A 2-cm longitudinal incision was made in the investing fascia 0.5 cm from the medial or lateral edge of the sternocleidomastoid muscle, and then blunt separation using fingers was performed between the deep surface of the sternocleidomastoid muscle fascial sheath and the carotid sheath. During separation, the degree of difficulty in separating the fascial sheath of the sternocleidomastoid from the carotid sheath was assessed, and separation continued until the lateral or medial edge of the sternocleidomastoid muscle was reached. A 1- to 2-cm separation toward the distal and proximal ends of the sternocleidomastoid muscle and the fascial sheath from the previously separated site was performed, and then the sternocleidomastoid muscle was divided but not removed. The head and neck were manipulated to observe the presence of residual tensioned lateral bands. The carotid sheath and anterior scalene muscle were then examined for contracture, and if present, the contracture site was divided (Fig. 2-A, B, C).

Neck immobilization was not performed in all patients after surgery. After the surgical pain subsided, the patients started moving the neck appropriately (similar to the intraoperative range of motion) to the opposite side of the preoperative deformity. The average follow-up period was 15 (1 to 24) months.

### 2.4 Methods of analysis

The motion (lateral bending and rotation) of the cervical spine, the head tilt angle, cosmetic and functional satisfaction, and the presence of lateral bands were assessed before surgery and at the end of the follow-up period. The head tilt angle was evaluated using the method described by Shim JS^[6]^; that is, the angle (cervical-mandibular angle [CMA]) was measured between the upper-edge connecting line of the 7th cervical vertebrae and the connecting line of the bilateral mandibular angle on the anteroposterior view of a radiograph. A CMA <5° was defined as excellent, a CMA between 6° and 10° was defined as good, a CMA between 11° and 15° was defined as acceptable, and a CMA > 15° was defined as poor. Neck motion and cosmetic and functional satisfaction were measured and assessed using the method described by Staheli.^[10]^

## 3 Results

### 3.1 Anatomical Studies

The investing fascia in the anterior neck is dense and completely envelopes the sternocleidomastoid muscle. The platysma muscle and the investing fascia can be easily and completely separated.

**Figure 3:**
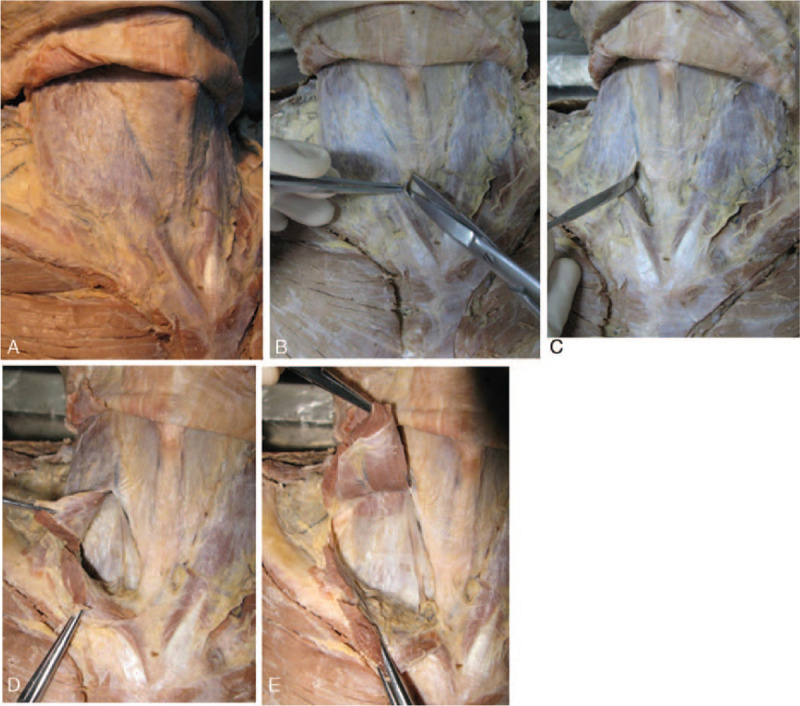
Anatomy of the fascial sheath of the sternocleidomastoid and its relationship with the carotid sheath. Fig. 1-A shows that after the platysma muscle is separated and retracted, the intact investing fascia can be visualized clearly, and complete separation from the platysma muscle is easy. As shown in Fig. 1-B, the investing fascia was incised in the medial edge of the sternocleidomastoid muscle. In Fig. 1-C, separation continues posterior to the sternocleidomastoid fascial sheath, and in Fig. 1-D, further lateral separation continues posterior to the fascial sheath of the sternocleidomastoid until the lateral edge of the sternocleidomastoid muscle is reached. Finally, Fig. 1-E shows that when the sternocleidomastoid muscle and its fascial sheath were divided and retracted, the deep structures, that is, the carotid sheath and the scapula geniohyoid muscle, were clearly visualized. The intact, dense fascia can be visualized in the transection of the divided sternocleidomastoid muscle and isolated from the carotid sheath.

In the middle and lower cervical sites, the posterior sheath of the sternocleidomastoid muscle and the carotid sheath are isolated and can be easily separated. When the sternocleidomastoid muscle and its fascial sheath were retracted, the deep structures, that is, the carotid sheath and the scapula geniohyoid muscle, were clearly visualized. The intact, dense fascia can be visualized in the transection of the divided sternocleidomastoid muscle and is not closely connected to the deep tissue. No significant difference was identified between the left and right sides (Fig. 3 A-E).

### 3.2 Clinical researches

All procedures were successfully completed, with an average operative time of 24 (18 to 27) minutes. Only oozing hemorrhage in the operative field but not active bleeding was observed. Two patients underwent division of the clavicular head. One patient underwent division of the sternal head. The remaining patients underwent division of bilateral muscle heads. The space between the fascial sheath of the sternocleidomastoid and the carotid sheath was accessible in all patients. During blunt dissection to the posterior-middle of the fascial sheath of the sternocleidomastoid, resistance is encountered in most procedures, but dissection can continue by slightly increasing the separation force. In 2 patients, the resistance during blunt dissection was relatively strong, and the operation was successfully completed by separating and dividing the sternocleidomastoid muscles from the medial and lateral sides. After the sternocleidomastoid muscle and its fascial sheath were divided, the carotid sheath was clearly visualized. In one patient, a thicker carotid sheath with tension was noted, which affected the rotation of the cervical spine, but the sheath was easily isolated and divided (Fig. 2-C). Scalene muscle tension was not observed in any of the patients in this study. Incision effusion occurred in one patient after surgery; the wound was reclosed by suture after 4 days of drainage and healed well. No postoperative complications were reported for the remaining patients.

**Figure 4:**
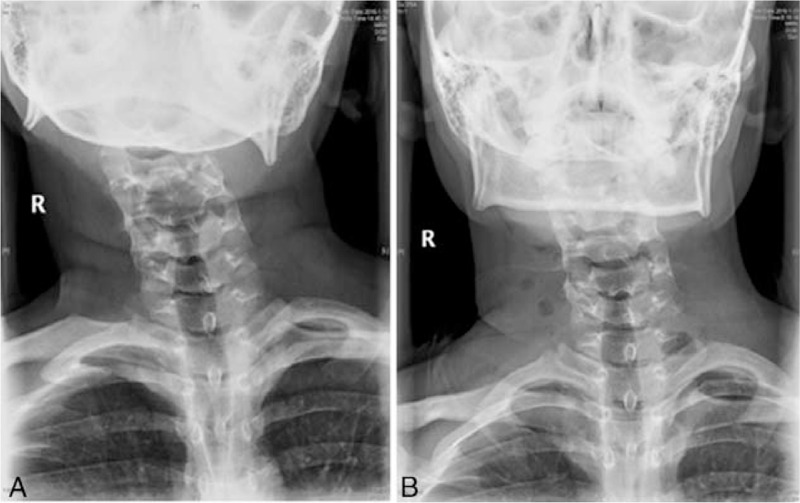
Fig. 4-A and Fig. 4-B show anteroposterior views of a patient's radiographs before and after surgery, respectively.

**Figure 5:**
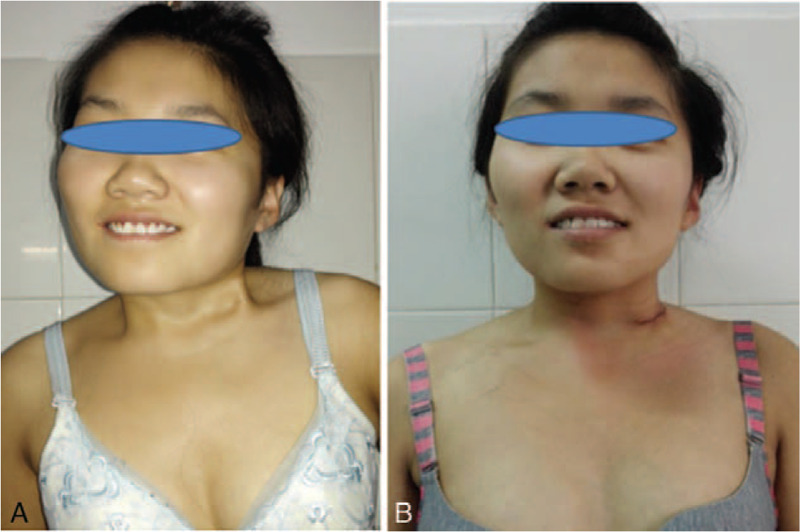
Fig. 5-A and Fig. 5-B show preoperative photographs and postoperative photographs of a patient, respectively.

The average follow-up period was 19 months (range: 5 to 43 months). The mean angle of lateral bending restriction was improved by 16.5° (19.3° preoperatively vs 2.8° postoperatively), and the mean angle of rotation restriction was improved by 5.1° (7.7° preoperatively vs. 2.6° postoperatively). The CMA was 19.6° (4.5°-31°) preoperatively and 2.3° (0°-10.6°) postoperatively, reflecting an average improvement of 17.3° (Fig. 4 A, B). According to the postoperative CMA, postoperative outcomes were considered excellent in 16 patients and good in 6 patients. No postoperative lateral band was noted in the neck in any of the patients. Cosmetic and functional satisfaction was excellent in 13 patients, good in 7 patients, and fair in 2 patients (Fig. 5 A, B).

## 4 Discussion

The mechanism of CMT involves either a soft tissue etiology or a bony etiology. Before puberty, when the body has not yet entered the rapid growth phase, CMT is mainly believed to be caused by a soft tissue etiology. In adulthood, the bony etiology predominates as the major cause of CMT, which cannot be corrected even after fully releasing the soft tissue tension because bony deformities cannot be corrected. However, soft tissue correction has been shown to significantly improve cosmetic appearance and head and neck motion.^[16,20]^ Our current study evaluated the anatomical relationship between the fascial sheath of the sternocleidomastoid and the carotid sheath, which is the base of our new designed surgery approach for the treatment of CMT. Anatomical observation in cadavers and clinical practical application revealed the feasibility and safety of the novel approach, which showed a significant advantage in reducing or eliminating postoperative bands compared with conventional surgical approaches.

Currently, surgical treatment of CMT mainly involves soft tissue correction. Soft tissue correction includes unipolar or bipolar sternocleidomastoid release and partial sternocleidomastoid muscle resection. Unipolar release is the most common procedure and can be applied alone or in combination with other procedures with proven effectiveness. The effectiveness of CMT correction is affected not only by the timing of correction but also the degree of the release of soft tissue contracture during surgery. Incomplete correction may or may not be perceptible after surgery. A lateral band is a perceptible indicator of incomplete correction of CMT. The lateral band may be the incompletely divided tendon structure of the sternocleidomastoid muscle or residual fascial sheath. Separation and division is easier to perform in the sternocleidomastoid muscle tendon but difficult in the posterior fascial sheath because the posterior sheath of the sternocleidomastoid muscle is adjacent to the carotid sheath. In conventional unipolar sternocleidomastoid release, isolation of the sternocleidomastoid fascia posterior sheath from the carotid sheath is difficult, and a risk of injury to the common carotid artery and especially the internal jugular vein, which are within the carotid arterial sheath, exists when the posterior sheath of the sternocleidomastoid fascia is divided. Therefore, the reasons for ineffective treatment of CMT using conventional surgical methods are related to both anatomical structures and the surgical approach.

Some incomplete correction of CMT may be imperceptible, which is due to complex anatomic and surgical reasons. In the pathogenesis of CMT, in addition to contracture of the sternocleidomastoid muscle and its fascial sheath, carotid sheath and/or scalene contracture may also be observed, which may be particularly important in patients with a long history and severe symptoms of CMT. Because both of these structures are deeply located, an incompletely divided contracture may not be visualized but still affect surgical effectiveness. In conventional unipolar sternocleidomastoid release, if the posterior sheath of the sternocleidomastoid muscle cannot be clearly visualized and divided, then exposure of the carotid sheath and the scalene muscle is impossible. If contracture of the carotid sheath and/or the scalene muscle cannot be treated at the time of complete division of the sternocleidomastoid muscle and the posterior sheath, the degree of motion of the cervical spine may be limited even if no lateral band can be visualized, reflecting another possible cause of ineffective surgical treatment of CMT. This scenario is easily overlooked but may occur frequently.

The above analysis indicates that the close relationship between the posterior sheath of the sternocleidomastoid and the carotid sheath is a key factor influencing the therapeutic effect of CMT treatment. Doctors cannot change the anatomy of the human body but can modify the surgical method. Compared to the procedure within the fascial sheath of the sternocleidomastoid in traditional unipolar release to correct CMT, the procedure in the site posterior to the sternocleidomastoid fascia posterior sheath, that is, separation between the sternocleidomastoid fascia posterior sheath and the carotid sheath, followed by division of the sternocleidomastoid muscle and its fascial sheath, can theoretically avoid or reduce the possibility of damaging the carotid sheath and its contents. However, a common assumption is that no tight junction exists between the fascial sheath and the carotid sheath, or it can be easily separated even if present. In this study, the anatomical study confirmed the presence of only loose connective tissue and no tight junction between the sternocleidomastoid fascial posterior sheath and the carotid sheath, which can be easily separated by blunt separation. Our clinical study proved that our novel surgical approach is feasible and can be used to separate the posterior sheath of the sternocleidomastoid and the carotid sheath. In contrast to the high incidence (38–75%) of postoperative lateral bands reported in the literature,^[4,5,13–15]^ no postoperative lateral band was observed in the neck in any of the patients, which is probably the main reason for the superior treatment effect observed in this study. Although this was a small clinical study, carotid sheath contracture was identified in 1 patient and was successfully treated. Imagining the use of traditional surgical methods to reveal the carotid sheath so clearly and to treat it safely is difficult.

The limitations of this clinical study are as follows: The novel approach used in this study was only preliminarily applied in a small population, and no control group (using a conventional approach) was available. The comparison between this current approach and other techniques will be also needed in our future work. The follow-up period was too short, and a longer period is needed to assess the correction of head and facial malformations. Consequently, we could only observe improvements in lateral bending, rotation, and the CMA, which are commonly used to assess a new surgical approach. However, we cannot assess improvements in cervical and facial deformities using any of the major overall evaluation systems. Moreover, the timing of correction was suboptimal for the older patients, and their outcomes may not adequately reflect the effect of this surgical approach. However, the author focused more on patient satisfaction among the older patients to determine the resultant ease and comfort associated with cervical spine mobility.

## 5 Conclusion

In summary, our current study observed the anatomical relationship between the fascial sheath of the sternocleidomastoid and the carotid sheath. In addition the separation of the sternocleidomastoid fascial sheath and the carotid sheath were performed for the treatment of the CMT patients (≥6 years old). We found that the posterior sheath of the sternocleidomastoid muscle can be easily separated and safely and completely divided, and the carotid artery sheaths as well as scalene muscles can be clearly visualized, and contractures can be easily managed. Follow-up and prognosis showed the feasibility and safety of this novel approach. Therefore, we recommended this surgical approach for the CMT treatment.

## Author contributions

**Data curation:** Heng Jiang, Zhicheng Zhang.

**Formal analysis:** Yang Zhang, Zhicheng Zhang.

**Methodology:** Heng Jiang, Yang Zhang, Zhicheng Zhang, Dajiang Ren.

**Software:** Bing Zhu.

**Supervision:** Jianlin Shan.

**Writing – original draft:** Jianlin Shan, Heng Jiang, Dajiang Ren.

**Writing – review & editing:** Jianlin Shan, Bing Zhu.

Jianlin Shan orcid: 0000-0002-0735-3410.
